# Designing and Evaluating Digital Mental Health Interventions: Scoping Review

**DOI:** 10.2196/77038

**Published:** 2026-04-29

**Authors:** Sarah Zainab Mbawa, Roelof Anne Jelle de Vries, Luciano Cavalcante Siebert, Koen van Turnhout, Willem-Paul Brinkman

**Affiliations:** 1 Interactive Intelligence, Department of Intelligent Systems, Delft University of Technology, Van Mourik Broekmanweg 6, Delft, 2628 XE, The Netherlands, +31 884818181; 2 Human Experience and Media Design, Digital Business and Media, University of Applied Sciences Utrecht, Utrecht, The Netherlands

**Keywords:** digital interventions, mental health care, design principles, evaluation approaches, guidelines

## Abstract

**Background:**

The ongoing adoption and use of digital interventions offer promising opportunities to meet the growing demand for mental health support. The effectiveness, implementation, and usage of these interventions depend on how well they are designed and evaluated. However, given the emerging nature of design research in this area, there is still no clear consensus on the specific principles and guidelines for developing digital mental health interventions (DMHIs). There seems to be a lack of clarity regarding the best practices for designing and evaluating these tools.

**Objective:**

We aimed to investigate and report on the design principles and evaluation approaches used in digital interventions specific to mental health care. Additionally, we sought to outline how these principles and approaches are applied in research.

**Methods:**

This scoping review was conducted in accordance with the PRISMA (Preferred Reporting Items for Systematic Reviews and Meta-Analyses) guidelines for scoping reviews. The literature search was performed in 2 electronic databases, SCOPUS and Web of Science, across 3 iterations from January 2024 to January 2025. A total of 2 independent reviewers screened and selected papers based on predefined inclusion and exclusion criteria, followed by data extraction from the selected studies. The data were then synthesized by categorizing the papers according to the primary research aim of each study. The inclusion criteria covered studies involving populations with mental health challenges or users of DMHIs, any digital tools for mental health care, and principles or strategies related to the design, evaluation, or implementation of DMHIs.

**Results:**

Our search identified 401 papers, of which 17 met the inclusion criteria for this review. Among these, 11 focused on evaluation studies, while 6 covered both design and evaluation studies (mixed). An iterative user-centered development process, expert inclusion, usability testing, specification of design elements, and user tracking and feedback were identified as common design principles used in studies focused on DMHIs. Evaluation approaches were shaped by the evaluation goal, which influenced the chosen methodologies. We also summarize the recommendations for implementation highlighted in some studies. Based on our findings, we propose 8 guidelines emphasizing stakeholder involvement in the development process and the need for clear justifications for design decisions, among other considerations.

**Conclusions:**

Design principles used in DMHI development include user-centered development, expert inclusion, and usability testing, while evaluation approaches often rely on randomized controlled trials to assess efficacy. Qualitative and mixed-method approaches are commonly adopted by studies to capture user experience and bridge both process and outcome measures. We recommend that future research explicitly report its design justification and adopt a multiperspective approach in the research and design of DMHIs.

## Introduction

### Background

Coping with the stresses of life, realizing one’s ability, learning, working, and contributing to the community rely on people’s state of mental well-being, often referred to as mental health [1]. However, attaining this state of mental health remains a common challenge for over 1 billion of the world’s population [2,3]. Thereby presenting a tremendous societal burden in terms of morbidity, quality of life, and premature mortality among others worldwide [3,4]. A range of evidence-based approaches have been developed and globally adopted to address these mental health conditions and are commonly regarded as biomedical, psychotherapeutic, and lifestyle-based interventions [5-7]. Biomedical treatments include pharmacological (medications) interventions such as antidepressants, mood stabilizers, and antipsychotics, and neuromodulation techniques, typically adopted for severe or treatment-resistant cases [5,6,8,9]. Psychotherapeutic treatments such as psychosocial (individual, group, or family) and cognitive-behavioral–based therapies are widely recommended as first-line treatments for common mental health conditions [5,6,8]. Lifestyle-based interventions, including exercise, mindfulness, and social support, have shown positive effects as supplement treatment approaches [7,10,11]. Additionally, studies recommend that combining different treatment approaches yield the most effective outcomes for mental health [8,11-13]. These interventions typically depend on in-person delivery, and while individuals experiencing mental health challenges want some form of treatment [14-16], multiple barriers hinder this [16]. These barriers include limited access to treatments, geographical and financial constraints, underresourced health care services [17], and stigmatization [18], which results in a high increase in mental health complaints. Accordingly, the rising incidence of mental health challenges causes a significant increase in the demands on health care systems, exceeding the available resources [19,20].

Given these limitations, technological interventions have emerged as promising supplementary solutions for their potential to enhance the scalability, affordability, and accessibility of mental health care [17,21]. Among these technological interventions are digital mental health interventions (DMHIs), which are nonpharmacological, often therapy-oriented, and lifestyle-supportive tools [22-24]. They are commonly delivered via digital platforms, such as the internet, smartphone apps, SMS, and virtual reality, aimed at preventing or alleviating mental health conditions [23,25,26]. DMHIs often use techniques such as cognitive behavioral therapy (CBT) or positive psychology [26] and are applied in both clinical and general populations [27-29]. Examples include artificial intelligence–based virtual agents for mental health care [30,31], digital platforms for early interventions in young people with mental health challenges [32,33], mobile health interventions for suicide prevention [34], virtual reality psychotherapy [35,36], and internet-delivered CBT [37]. Despite their potential, designing and implementing DMHIs present significant challenges. These include the lack of personalization, limited human resources, technical and ethical considerations, and difficulties in clinical integration [38]. Additionally, applying suitable evaluation strategies remains a complex task [39,40], further complicating the development and assessment of effective interventions. Existing standards [41] (eg, ISO [International Organization for Standardization] 9241, 2019) and guidelines [42] (eg, Interaction Design Foundation, 2015) for digital technology design and evaluation are often field-specific, making them difficult to translate across different disciplines [40]. While identifying design principles is more common in human-computer interaction (HCI), it is less frequent in clinical science [43].

Efforts are being made to derive design principles for DMHIs from learning theories, such as repeated testing, interleaving, and spacing [44], as well as adapting HCI principles to formulate guidelines [45]. Doherty et al [45] emphasized that human-centered design (HCD) approaches, such as user-centered design and participatory design, can be adapted for mental health care technologies. They proposed guidelines such as designing for desired outcomes, collaborating with mental health professionals, adapting user-centered design for health care settings, and refining both system protocols and design during development. Evaluation guidelines included multiple stages of testing, evaluating with nonclinical users, using therapists as proxies, and monitoring unsuccessful cases. While these guidelines [45] and principles [44] offer a foundation for future DMHI design, Murray et al [39] noted difficulties in building a consistent knowledge base for evaluating digital health interventions (DHIs). Rapid technological evolution, gaps between research and publication, and varying patients’ needs limit the usefulness of current guidelines for supporting design decisions. Michie et al [40] further highlighted the need for scientific principles to guide DMHI design, evaluation, and implementation in health care, urging interdisciplinary collaboration to advance research methods.

Hrynyschyn et al [46] conducted a scoping review of evaluation methods beyond randomized controlled trials (RCTs) for DHIs. They found that factorial designs, stepped-wedge designs, sequential multiple assignment randomized trials, and microrandomized trials are common approaches. These methods allow for intervention adaptation and component evaluation, yet challenges remain in establishing these approaches in research practice and addressing their limitations, particularly within collaborative design processes in mental health care. Similarly, Balcombe and De Leo [38] focused on identifying the evaluation of digital mental health (DMH) platforms used and DMHIs applied on the DMH platforms. Their report highlighted the feasibility of DMH platforms and DMHIs, although the evidence for their effectiveness, quality, and usability is mostly heterogeneous and preliminary. In the context of design principles, Vial et al [47] reported that attempts have been made to integrate HCD approaches into DMHI development in their exploratory mapping review. Nevertheless, these approaches rely very little on designers and design research. They provided suggestions for better reporting of HCD approaches in future research. These include (1) stating and defining the HCD approaches used in the design process and explaining why it was used, (2) describing the core elements of HCD activity, defining the steps and methods used, and explaining the extent to which actors were involved in the design process, and finally, (3) indicating the number of designers involved in the design process, their design profession, and the manner of their contributions.

### Research Aim

While previous research [38,46,47] has explored the existing design principles and evaluation approaches for DMHI, there is a need to understand how these concepts are applied in research and design. Improved access to and understanding of design principles and evaluation approaches could enhance DMHI development, foster stakeholder collaboration, and lead to more effective implementation strategies [48,49]. Therefore, our study aims to review existing principles and approaches used in DMHI design and evaluation, providing an overview of their application and implications using a scoping review approach.

For this study, a principle refers to fundamental guidelines or frameworks derived from interdisciplinary knowledge, offering process guidance to improve the likelihood of successful DMHI development [42,50]. We refer to the methods or strategies used to evaluate DMHIs as an “approach.” Textbox 1 provides a description of what we mean by the terms design and evaluation.

Textbox 1. **Definition of the terms used in this study.**

- Design: the design or development process of an application or digital intervention for mental health care, whether a proof of concept or a fully functional intervention.
- Evaluation: examining or investigating the effectiveness, engagement, user experience (UX), usability, functionalities, and performance of any digital intervention for mental health care.

Our review makes 2 important contributions. First, we provide a comprehensive review of the principles and approaches used in the design and evaluation of DMHIs. Second, we propose 8 guidelines for DMHI design and evaluation based on the results of this review. Although we initially aimed to explore implementation strategies, none of the identified studies explicitly focused on DMHI implementation. Therefore, we concentrated on identifying implementation strategies recommended by the reviewed literature.

The research questions guiding this review are as follows: (1) What design principles and evaluation approaches are used in DMHIs? (2) How are these principles, approaches, or strategies applied in DMHIs development process?

## Methods

### Study Design

The field of DMHI is relatively new; therefore, our research focuses on providing a review of existing principles and approaches for designing and evaluating DMHIs. This meant that a variety of study designs would be included in our review; a scoping review is most appropriate for this study [51]. We followed widely accepted guidelines for reporting a scoping review: JBI (Joanna Briggs Institute) Scoping Review Methodology [52] and the PRISMA-ScR (Preferred Reporting Items for Systematic Reviews and Meta-Analyses extension for Scoping Reviews) guidelines [53].

### Inclusion and Exclusion Criteria

We designed the inclusion criteria to align with the research objectives of our study, which aims to provide an overview of how existing design principles and evaluation approaches are applied in the design and evaluation of DMHIs. The general idea of the inclusion criteria was to include only primary empirical studies reporting on the design, implementation, or evaluation of DMHIs. In addition, we defined the exclusion criteria in Textbox 2.

Textbox 2. **The exclusion criteria used for this study.**

- Type of publications: reviews, commentaries, letters to the editor, meta-analyses, literature studies, Delphi studies, framework developments, and conference abstracts.
- Papers that only present study protocols, but do not present the execution and results.
- Studies focused on conditions other than mental conditions as a primary condition, or never explicitly defined the mental health condition.
- Studies that do not explicitly mention interventions that are completely digital or internet-based but use digital tools only for distribution or recruitment instead of implementation or intervention.
- Papers that do not explicitly mention or report on any design or study or implementation or evaluation of a DMHI.
- No (explicit) mention, description, or use of a principle, framework, strategy, or guideline in designing, implementing, or evaluating a DMHI.

Following JBI guidance and the PRISMA-ScR reporting standards, studies included in our review had to fulfill the following Population, Concept, and Context criteria:

(a) Population:

- Studies involving individuals or groups experiencing any type of mental health challenges, conditions, or illnesses (eg, depression, psychosis, anxiety, etc), irrespective of age, gender, or cultural background.
- Studies targeting users of digital interventions aimed at mental health promotion, prevention, treatment, or well-being from the general population.
- No restrictions were applied to specific populations (eg, clinical vs nonclinical) provided that the intervention or study focus addressed mental health outcomes explicitly.

(b) Concept:

- Studies that design, develop, implement, or evaluate any type of DMHI (eg, mobile apps, online therapy platforms, chatbots, web-based self-help tools, or virtual reality interventions).
- Included studies must explicitly reference, describe, or apply a principle, theoretical framework, model, strategy, or guideline during any phase of the DMHI lifecycle (including design, implementation, or evaluation).
- Eligible studies may use qualitative, quantitative, or mixed-methods approaches, and report on engagement, usability, effectiveness, or implementation outcomes.

(c) Context:

- Studies conducted in any geographical, cultural, or health care context, including community, clinical, educational, and workplace settings.
- The context must clearly involve mental health care, treatment, or well-being, ensuring the digital intervention is applied within a mental health objective.

### Search Strategy

We used SCOPUS and the Web of Science database to search for relevant literature to be included in this study. The search was conducted from January 2024 to February 2024, with only English-language journal papers and conference papers published. There were no limitations on the year of publication. We ran another search in January 2025 on the same databases and inclusion and exclusion criteria as before, but no new papers were included in the review.

### Search Terms

The search structure combined appropriate keywords and controlled vocabulary terms for 5 concepts: DHIs, design, implementation, evaluation, and mental health. We checked previous literature reviews [38,39,46,47] to validate these terms. We based these search terms on the target intervention (eg, digital or online health interventions), condition (eg, mental disorder, depression, stress, anxiety, etc) and the research or project activity (design, implementation, and evaluation). A detailed overview of the search strings used for searching the Scopus database can be found in Multimedia Appendix 1.

### Selection of Sources of Evidence

All results were exported to Excel (Microsoft Corp) and Mendeley (Elsevier.com) reference management software for deduplication. The exported CSV files were then imported into Excel for title, abstract, and full-text screening. Two authors independently screened the selected studies based on title or abstract and resolved any discrepancies by consensus during discussions. Cohen κ was calculated to assess the intercoder agreement between the inclusion and exclusion codes, which showed an excellent agreement (0.81) for screening titles and abstracts. A list of papers included and excluded after full-text screening is presented in Multimedia Appendix 2 [54-70].

### Data Items

The following data were extracted from the selected studies:

- Bibliographic information: title, first author, year of publication, and country.
- Study and participant characteristics: study design, study type, sample size, and age of participants.
- Characteristics of the digital intervention: type, name, purpose, targeted disorder, and features.
- Design, evaluation, and implementation strategies: design principles, evaluation approaches (including methods, tools, outcome measures, and data collection techniques), and implementation strategies for the intervention.

### Data Synthesis

The data were divided into groups based on paper type; a code for whether the study aim was either a combination of design (development) and evaluation, or solely focused on evaluation of a DMHI (henceforth referred to as design and evaluation studies, and evaluation studies, respectively). This provided a more concise approach to reporting the relevant principles or guidelines used for the research activity or paper type, as studies that were focused on DMHI design used different principles than those focused on evaluating a DMHI.

## Results

### Search Results

**Figure 1.**
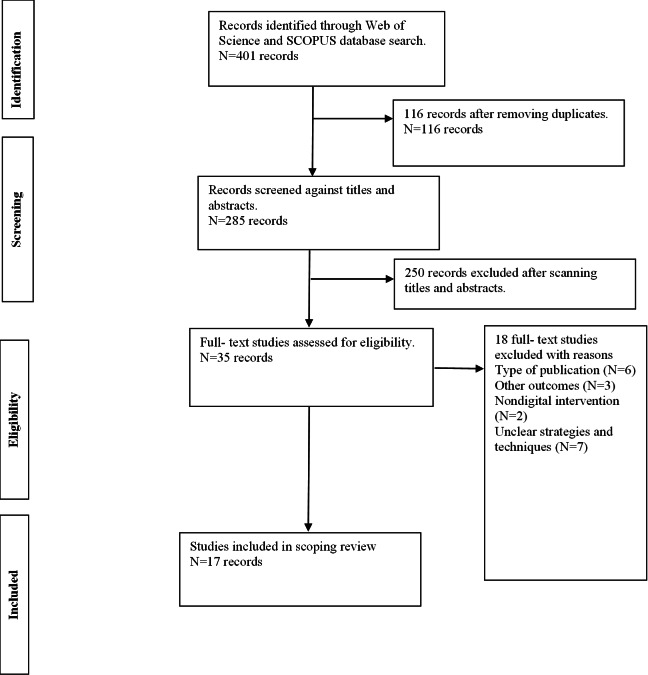
inPRISMA flow diagram of the study process. PRISMA: Preferred Reporting Items for Systematic Reviews and Meta-Analyses.

A total of 401 papers were identified across the Scopus (193 papers) and Web of Science (208 papers) databases, with 116 duplicates removed. The titles and abstracts of the remaining 285 papers were screened based on the exclusion and inclusion criteria, leading to the exclusion of 250 papers. Full texts of the remaining 35 papers were then downloaded and assessed against the inclusion and exclusion criteria. After reviewing these 35 papers, 18 were excluded, resulting in 17 papers being included in this review. These papers highlighted the design principles, evaluation approaches, and design processes. Figure 1 illustrates the selection process of the studies.

### Study Characteristics

Table 1 provides an overview of the 17 papers included in the review (see Multimedia Appendices 3-5 [43,71-86] for a detailed overview). The studies reported in the papers were published between 2011 and 2023 and were conducted in Europe (6 papers), North America (6 papers), Australia (2 papers), Asia (1 paper), Africa (1 paper), and 1 paper in both Europe and Africa. Of the 17 papers, 11 papers focused on evaluation studies, and 6 papers covered both development and evaluation studies. In terms of study design, 8 were qualitative studies, 2 used mixed methods, and 7 were quantitative studies—with 3 RCTs, 2 retrospective observational studies, and 2 unspecified. Most digital interventions were mobile-based (7 papers), followed by web-based (5 papers), while the remaining (4 papers) were a combination of web and mobile-based interventions, with 1 paper being internet-based. The included papers will be discussed further based on their respective categories: design and evaluation (mixed) and evaluation.

**Table 1.** General characteristics of papers (n=17).

Author, year of publication; country^a^	Study type (paper type^b^^,^^c^)	Study design	Intervention type (name)
Xiang et al, 2023 [71]; United States	Development and comparative usability evaluation^b^	Qualitative	Web-based (Empower@Home)
Shkel J et al, 2023 [43]; United States	Evaluation of user experience and perception^c^	Qualitative	Web-based (Overcoming Thoughts)
van Orden et al, 2022 [72]; Netherlands	Evaluation of effectiveness and efficiency^c^	Exploratory naturalistic retrospective cohort study	Mobile and web-based (NiceDay)
Cuijpers et al, 2022 [73]; Lebanon	Evaluation of effectiveness^c^	Single-blind, 2-arm pragmatic RCT^d^	Mobile and web-based (Step by Step)
Harty et al, 2023 [74]; Ireland	Evaluation of effectiveness^c^	Quantitative retrospective observational study	Web-based (SilverCloud)
Kerber et al, 2023 [75]; Germany	Evaluation of effectiveness^c^	RCT	Mobile-based (Mind Doc)
Mayer et al, 2022 [76]; Germany	Evaluation of user experience and acceptance^c^	Mixed method cross-sectional observational study	Mobile and web-based (SELFPASS: Self-Administered Psycho Therapy Systems)
Burchert et al, 2018 [77]; Germany, Sweden, and Egypt	Adaptation process study^b^	Qualitative	Mobile-based (step by step)
Stegemann et al, 2013 [78]; Germany	Development process study^b^	Qualitative	Mobile and web-based (GET.ON PAPP)
Geraghty et al, 2016 [79]; United Kingdom	Development and evaluation of usability^b^	Qualitative	Internet-based (Healthy Paths through Stress)
Venkatesan et al, 2020 [80]; United States	Evaluation of effectiveness^c^	Quantitative retrospective observational	Mobile-based (Vida Health)
Ferguson et al, 2021 [81]; United States	Design and evaluation of engagement^b^	Mixed method	Mobile-based (The Guardian: Unite the realms)
Valentine et al, 2020 [82]; Australia	Evaluation of engagement and user experience^c^	Qualitative	Web-based (Horyzons)
Gould et al, 2021 [83]; United States	Evaluation of effectiveness^c^	Quantitative	Mobile-based (Meru Health program)
Graham et al, 2020 [84]; United States	Evaluation of efficacy^c^	RCT	Mobile-based (IntelliCare)
Klein et al, 2011 [85]; Australia	Evaluation of effectiveness^c^	Quantitative quasiexperimental	Web-based (AnxietyOnline)
Pozuelo et al, 2023 [86]; South Africa and Uganda	Development and usability^b^	Qualitative	Mobile-based (Kuamsha app)

a Country of the study setting.

b Design or development and evaluation study.

c Evaluation study.

d RCT: randomized controlled trial.

### Overview of Targeted Mental Health Conditions

Across the 17 papers included in this review, various mental health conditions were targeted in their subsequent studies. Depression was the most frequently targeted condition, investigated in 10 studies [43,71-73,76,77,80,83,84,86]. Anxiety was also commonly examined, appearing alongside depression in 5 papers [43,72,80,83,84] and in 1 study combined with other conditions such as obsessive-compulsive disorder, posttraumatic stress disorder, and panic disorder [85]. Panic disorder was also the primary focus of 1 additional study [78]. General mental well-being was explored in 3 studies [74,75,81], while psychosis [82] and emotional distress [79] were examined in 1 study each. A detailed overview of the included papers and their corresponding target conditions is provided in Multimedia Appendix 5.

### Design and Evaluation Studies

Among the 17 included papers, 6 studies focused on the design and evaluation of DMHIs. Two of these studies were qualitative and focused on design and development, while 4 additional studies used mixed methods approaches that included design and evaluation (mixed). An overview of these studies is presented in Table 2. All studies shared several common design principles, emphasizing user-centered and iterative design processes often involving co-design with users and expert consultation or collaboration to ensure the intervention was both effective and user-friendly. Usability testing was a key evaluation approach across studies, with feedback loops used to refine and improve the interventions. Additionally, the mixed studies incorporated a focus on behavioral engagement through features such as mood tracking, real-life exercises, and gamification elements. Due to the various design principles and evaluation approaches used in the included studies, we categorized these into 5 groups. Additionally, we reported the recommended implementation strategies highlighted in the studies. Table 2 illustrates an overview of the included studies.

**Table 2.** Design (development) and evaluation studies.

Study	Design principles for development and adaptation	Evaluation approaches	Recommendations	Recommended implementation strategies
Burchert et al, 2018 [77]	User-centered adaptation process Iterative prototyping Usability testing Contextual adaptation	Rapid appraisal	Mental health apps should provide more sessions in shorter intervals than web-based interventions. Ensure intuitive user interfaces, and provide a clear structure for less technically literate users. Improve motivation and engagement through interactivity. If contact-on-demand is used, it should happen with low response latencies. User testing with clinical cases. Iterations of prototype testing with users over a longer period in a natural environment.	Main barriers for implementation: Acceptability, credibility, and technical requirements.
Stegemann et al, 2013 [78]	Iterative development process Expert inclusions and collaboration Event-based design Minimal design Casual information visualization Mondrian-style display User feedback and tracking	Informal team testing	NR^a^	NR
Xiang et al, 2023 [71]	E-health development strategies: multidisciplinary team approach Iterative user-centered design process Co-design and passive storyboarding techniques using persuasive and emotional design Agile development process Accessibility and learning	Usability testing	Close collaboration with stakeholders Iterative design process Attention to user experience	Peer support on a therapeutic social network The number of log-ins does not reflect a young person’s experience of the intervention Unclear social protocol creates an uncomfortable social environment in the digital space Social anxiety, paranoia, internalized stigma, and the perception of limited autonomy could interrupt a young person’s ability to engage with the platform.
Ferguson et al, 2021 [81]	Character and narrative design Behavioral engagement Gameful play Immediate-use daily rewards Mood reflection Curated activities Escapist aesthetics Task management Long-term interest Gacha games influence	Public feedback approach	Consider variants of the game mechanic, narrative, and graphics	NR
Geraghty et al, 2016 [79]	Intervention logic model Inclusion of user perspective Explorative qualitative interviews, including think-aloud Person-based approach Prototyping	Usability testing by data analysis	Ensure diversity in sampling. Create recruitment materials that extend reach beyond the initial group. Provide and articulate therapeutic rationales for others to evaluate and determine their coherence.	NR
Pozuelo et al, 2023 [86]	Iterative user-centered agile design Co-design and extensive expert consultation Comprehensive development process: conceptualization, prototyping, product release, and evaluation Iterative feedback loop Storytelling and game design Accessibility and confidentiality	Usability testing: randomized controlled trial	App development work should explore integrating audio voice-overs or alternate features to enhance accessibility for populations with low literacy.	NR

a NR: not reported.

### Design Principles Reported in the Studies

We identified five groups of design principles in the studies reviewed: HCD approaches such as (1) user-centered design, (2) iterative development, (3) engagement and motivation, (4) design specificity, and (5) security and accessibility. Among these, the principles of engagement and motivation and iterative development were commonly used to enhance user experience and personalize interventions to the specific needs of target populations. These principles ensured that the designs were both relevant and adaptable. The reported design principles are detailed below.

Of user-centered design, all studies emphasized the importance of user-centered design, directly involving users and relevant stakeholders in the design process. We synthesized this aspect into 2 approaches as highlighted in the included studies: direct end user involvement and expert-driven collaboration.

First, for direct end user involvement, Burchert et al [77] performed interviews, focus groups, and usability testing with real or intended users (Syrian refugees), focusing on their needs and how users interacted with the design, including barriers to implementation in practice. Similarly, Xiang et al [71] and Pozuelo et al [86] adopted co-design methods, engaging users as active collaborators in the design process. This approach emphasized accessibility, learning, and iterative refinement driven by user feedback. Geraghty et al [79], on the other hand, used explorative qualitative interviews to capture user perspectives, adopting a person-based approach to ensure that the intervention aligned with users’ needs and contexts.

Second, for expert-driven collaboration, Stegemann et al [78] prioritized expert inclusion and informal team testing, suggesting a collaborative process focused on expert knowledge as a guiding influence for their design process. Pozuelo et al [86] also consulted experts during co-design but maintained a user-centered approach by including users in the co-design and iterating their prototype based on user feedback. Ferguson et al [81] used a theory-centered design, involving focus groups with users to inform character and narrative design. Their approach balanced behavioral engagement features with user involvement to refine prototypes.

Of iterative development, all studies adopted iterative development processes, focusing on continuously refining designs based on feedback. Studies relied primarily on either end user feedback or on expert-driven iteration.

First, for end user feedback, Burchert et al [77] used iterative prototyping and usability testing with the target users, allowing direct feedback to shape subsequent versions. Similarly, Xiang et al [71] and Pozuelo et al [86] adopted agile methods, iteratively refining their digital intervention based on user input. Geraghty et al [79] focused on prototyping, integrating user feedback into successive iterations to improve the intervention’s relevance and functionality.

Second, for expert-driven iteration, Stegemann et al [78] emphasized informal team testing and expert collaboration in their iterative process. This approach leaned on expert input rather than direct user interaction for refining prototypes. Ferguson et al [81] combined task management principles with adaptive design, allowing game features to evolve during user interaction but supported by expert oversight and theoretical frameworks such as behavioral engagement.

Of user engagement and motivation, the studies adopted varied approaches to enhance user engagement and motivation. These can be synthesized into 2 main strategies: user-centered and behavioral design, and gamified or emotional engagement.

First, user-centered and behavioral design, Burchert et al [77] used the Integrate, Design, Access, and Share framework, combining usability testing with free list interviews, key informant interviews, and focus group discussions to ensure the tool met users’ needs. This approach focused on tailoring the design to align with users’ behavior and preferences. Geraghty et al [79] used a person-based approach, focusing on intrinsic motivation by offering users choices and avoiding directive or medicalized language to foster a sense of autonomy and engagement.

Second, gamified or emotional engagement, Xiang et al [71] integrated persuasive and emotional design elements, such as motivational quotes and animated storytelling, to create a deeper connection with users. Pozuelo et al [86] and Ferguson et al [81] incorporated gamification strategies, such as storytelling, mood reflection, and immediate-use rewards, to sustain user motivation and encourage long-term participation. Stegemann et al [78] included functions that allowed users to track their behaviors by documenting panic-related events or daily summaries of their state and progress and providing feedback. They adopted a more data-driven approach to engagement by interpreting user actions rather than direct involvement in the design.

Of design specification, the studies varied in their emphasis on general versus specific design elements, with 2 studies highlighting the balance between general contextual adaptation and specific aesthetic design choices.

First, for general contextual adaptation, Burchert et al [77] emphasized contextual adaptation as a core principle, focusing on aligning the intervention with users’ cultural and situational contexts. Their approach prioritized the overall process over detailed interface elements, ensuring flexibility in design to suit diverse user needs.

Second, for specific aesthetic design choices, Stegemann et al [78] presented highly specific design choices, such as a Mondrian-style display, event-based design, minimal interface design, and casual information visualization. These choices focused on functional simplicity and an aesthetically pleasing user experience, prioritizing clarity and ease of interaction.

For security and accessibility, 2 studies addressed these through 2 approaches: designing for ease of use and ensuring data confidentiality.

First, for designing for ease of use, Xiang et al [71] prioritized accessibility by incorporating large buttons, text descriptions for icons, high-contrast color schemes, and intuitive navigation. These features aimed to ensure usability for a broad demographic, including older adults and individuals in low-resource settings. Pozuelo et al [86] extended accessibility by offering the intervention both online and offline, ensuring that users with limited internet access could still benefit from the tool.

Second, ensuring data confidentiality, Pozuelo et al [86] also incorporated security measures, such as password protection and an emergency button, to safeguard sensitive mental health data.

### Categories of Evaluation Approaches

The included studies focused on evaluating the engagement and user experience of their respective interventions. We categorized the various evaluation approaches that were used for evaluation into three groups: (1) usability testing, (2) qualitative feedback and focus groups, and (3) iterative feedback and adaptation.

All studies used various usability testing approaches that were dependent on the study or research at hand, creating a distinction between user-centered testing and internal or indirect testing.

First, for user-centered testing, Xiang et al [71] and Geraghty et al [79] conducted direct usability testing with target users during the development phase to ensure the design met user needs. Pozuelo et al [86] used RCTs to collect structured feedback from participants, allowing for an evaluation of usability. Burchert et al [77] implemented rapid appraisal techniques, including focus groups with the intended users, to verify findings and refine the iterations of their prototype.

Second, for internal or indirect testing*,* Ferguson et al [81] focused on feedback analysis after the public release of their intervention, emphasizing long-term user engagement and adaptation. Stegemann et al [78] focused on informal team testing, relying on internal feedback rather than direct user input during development.

For qualitative feedback and focus groups, qualitative feedback was a common approach used for understanding user experiences, with studies using either exploratory interviews or participatory workshops as their primary approach. These methods illustrate how qualitative feedback supports an in-depth understanding of user perspectives, enabling iterative refinement based on user experiences.

First, for exploratory interviews, Geraghty et al [79] used think-aloud methods and explorative qualitative interviews to gather in-depth insights into user interactions and preferences.

Second, Pozuelo et al [86] combined participatory workshops with focus groups to evaluate their intervention, involving users in collaborative sessions to refine the design.

For iterative feedback and adaptation, the studies adopted iterative feedback as a key approach for refining their intervention. This was, however, split between structured development loops and postrelease adaptation.

First, for structured development loops*,* Pozuelo et al [86] used an ongoing feedback loop during the development and evaluation phases, continuously refining the intervention based on user input.

Second, for postrelease adaptation*,* Ferguson et al [81] focused on engagement and feedback analysis after the public release of the app, allowing real-world user interactions to shape subsequent updates.

### Recommended Implementation Strategies

Although our initial goal included exploring implementation strategies, none of the identified studies explicitly focused on DMHI implementation. However, some studies did offer recommendations for implementing their interventions or highlighted barriers that could impact the implementation process. For instance, Xiang et al [71] reported on the possible implementation of their intervention in real-life settings (Table 2). Their results identified features for improving peer support and barriers that might influence the experience and engagement with their DMHI. Mental health condition, internalized stigma, and perception of autonomy could influence the users’ engagement, while the classification of users’ experience should not be dependent on the login frequency. Burchert et al [77], however, reported on the barriers that might influence the implementation of their intervention in real-life: acceptability, credibility, and technical requirements (Table 2).

### Evaluation Studies

Of the 17 papers reviewed, 11 focused on evaluating DMHIs using qualitative, quantitative, or mixed-method approaches. A total of 8 of the studies focused on evaluating the effectiveness of the intervention, and they typically used quantitative methods such as RCTs, retrospective observational studies, and quasi-experimental designs. Further, 3 studies specifically evaluated user experience, perception, and engagement of the DMHI using qualitative or mixed-method evaluation approaches. Table 3 presents an overview of the papers, including the evaluation approaches used in the studies. We further discuss the various evaluation approaches used in the included studies and the recommended implementation strategies reported in the following sections.

**Table 3.** Evaluation studies.

Study	Evaluation approaches (including methods and tools)	Recommendations	Recommended implementation strategies
Shkel et al, 2023 [43]	RCT^a^ Purposive sampling Semistructured interviews PHQ^b^ Repeated testing	Provide an option for users to share responses publicly or keep them private could be useful for future DMHIs^c^. Clearly defined target uses and evaluation metrics, as well as A/B testing and data-driven iteration, can improve their effectiveness and development.	Provide the toggle option to choose whether they want to complete the platform’s exercises privately or share their responses with others. Match users’ clinical profiles to appropriate DMHIs or guide when and how to use the DMHI effectively.
van Orden et al, 2022 [72]	KLANT (short) questionnaire Routine outcome monitoring Qualitative data	Research should compare DMHI and face-to-face treatment cost-effectiveness, explore patient readiness, establish guidelines for engagement during implementation, and assess the impact of digitalization of treatment on therapeutic alliance, outcomes, and relapse prevention.	DMHIs should offer personalized treatment by adapting steps and content to each patient’s needs. Timely registration and monitoring provide professionals with accurate, real-time data, improving session focus and supporting adherence to treatment plans.
Cuijpers et al, 2022 [73]	PHQ WHO^d^ Disability Assessment and WHO-5^e^ Well-Being Index GAD-7^f^ item Post-Traumatic Stress Disorder Checklist Diagnostic and Statistical Manual Psychological Outcomes Profile (PSYCHLOPS^g^) Client Satisfaction Questionnaire Outcome questionnaire	Further research is needed to provide an understanding of how to prevent or reduce dropout rates.	Implementation of the step-by-step through health authorities, local medical centers, and employers.
Kerber et al, 2023 [75]	RCT Baseline, postintervention assessment, and follow-up assessment Mental Health Literacy Questionnaire Assessment of Mental Health-Related Patient Empowerment Self-Management-Skills questionnaire Inventory of Attitudes Toward Seeking Mental Health Services Quality of Life-8 dimensions assessment Personality Inventory for DSM-5^h^, brief form plus Operationalized Psychodynamic Diagnosis-Structure Questionnaire Short	Early symptom improvement through self-guided digital interventions may support recovery and prevent chronic conditions. Research should explore their effects across health care settings, customize app behavior, content, and guidance based on personality or psychopathology, and provide increased guidance for users with severe psychopathology. Clinical interviews are needed to confirm diagnoses and assess the impact on mental health literacy and help-seeking, especially in treatment-naive primary care participants.	A longer intervention and follow-up period may be necessary to induce and measure changes in trust among mental health providers.
Mayer et al, 2022 [76]	Daily evaluation sheets PHQ-9	NR^i^.	Focus on the interaction between technology use and potential interventions to address depression. Recommendations for developers and clinicians when designing digital interventions include personalization, transparency, data security, serious design, gamification, structure, and crisis management.
Valentine et al, 2020 [82]	Open-ended questions Field notes Reflective log Phenomenological approach	Research should identify features and measures that improve engagement and minimize disruptions in long-term social media-based interventions for young people. Interviews should be conducted close to the end or during the intervention, as suggested by user design theory, to gather more relevant feedback.	While peer support can be helpful and engaging for some, it may be burdensome for others. However, shared experiences contribute to the creation of a social space. It is important to maintain an upbeat environment, as unclear social protocols can create discomfort. Potential interruptions such as internalized stigma and perceived lack of autonomy may affect participation and engagement.
Klein et al, 2011 [85]	e-PASS assessing for 21 DSM-IV-TR^j^ Kessler-6	Conduct RCT to compare all 5 e-therapy programs with waitlist controls and current best-practice face-to-face treatments. Investigate which individuals benefit most from e-therapy and develop strategies to improve adherence to the treatment protocol.	NR.
Venkatesan et al, 2020 [80]	PHQ-8 GAD-7 Biopsychosocial intake questionnaire	NR.	NR.
Graham et al, 2020 [84]	RCT PHQ-8 GAD-7 App use (log in) Number of messages sent	NR.	App usage preference and platform approach are dependent. Highlighting the need for future research to develop and evaluate strategies for integrating DMHI platforms into clinical workflows. Additional design efforts are needed to adapt these interventions for adolescents, given their high mental health needs.
Harty et al, 2023 [74]	PHQ-9 GAD-7 Service-level metrics such as referrals and account activations, user-level data, baseline symptomatology, clinical outcomes, program usage, and user satisfaction	Provide health professionals with information materials on SilverCloud suitability and referral guidance. Ensure eligibility, excluding those aged younger than 18 years or with severe symptoms. A designated supporter should offer feedback based on user progress and clinical symptoms.	NR.
Gould et al, 2021 [83]	Semistructured psychiatric diagnostic interview, PHQ Mini-neuropsychiatric Interview Psychosis screening questions, substantial alcohol use assessment, AUDIT-C^k^, Short-Blessed Test Suicide Risk Screener Cognitive and Affective Mindfulness Scale-Revised WHOQOL-BREF^l^ (UCLA^m^) Loneliness Scale (version 3)	NR.	Further consideration is needed for incorporating meaningful peer interactions in mental health interventions targeting older users. Additionally, interventions in controlled studies should be examined to better understand variations in intervention outcomes by age.

a RCT: randomized controlled trial.

b PHQ: Patient Health Questionnaire.

c DMHI: digital mental health intervention.

d WHO: World Health Organization.

e WHO-5: World Health Organization Well-Being Index.

f GAD-7: Generalized Anxiety Disorder-7 Scale.

g PSYCHLOPS: psychological outcome profiles.

h *DSM-5*: *Diagnostic and Statistical Manual of Mental Disorders, Fifth Edition.*

i NR:

j *DSM-IV-TR*: *Diagnostic and Statistical Manual of Mental Disorders, Fourth Edition, Text Revision*.

k AUDIT-C: Alcohol Use Disorders Identification Test-Concise.

l WHOQOL-BREF: World Health Organization Quality of Life.

m UCLA: University of California, Los Angeles.

### Evaluation Approaches Reported in Studies

The evaluation approaches reported by the studies can be categorized into two groups that influence the setup of a DMHI evaluation: (1) evaluation focus and (2) methodological approach (qualitative, quantitative, or mixed methods). These aspects highlight underlying decisions researchers face, in turn shaping the design and outcomes of their evaluations.

For evaluation focus, studies highlighted 4 areas on which DMHIs were evaluated. These areas include effectiveness, user experience, implementation feasibility, and longitudinal tracking. Providing insights into user engagement, clinical integration, sustained intervention use, and DMHIs’ impact on symptom reduction.

First, for effectiveness*,* 8 studies concentrated on evaluating the effectiveness of DMHIs, often using quantitative methods such as RCTs, retrospective observational studies, or quasiexperimental designs. These methods are suitable for assessing measurable outcomes such as symptom reduction or behavior change. In contrast, 4 studies explicitly evaluated user experience, perception, and engagement with DMHIs, using qualitative or mixed method approaches to gain deeper insights into user interactions and satisfaction. The evaluation focus of the experiment determined the type of methodological approach to be used. For example, studies focused on assessing the effectiveness of the intervention or for symptom tracking primarily used quantitative approaches. Further, 4 studies conducted RCTs to assess the intervention outcomes. Studies also used standardized tools and quasiexperimental designs to measure changes in mental health symptoms over time [73-75]. Other studies used retrospective observational designs to assess real-world effectiveness in uncontrolled environments [72,74,80].

Second, for user experience, 2 studies conducted user-centered evaluation by focusing on understanding user experiences, perceptions, satisfaction level, and engagement with DMHIs. This approach provides insights into the subjective experiences and contextual nuances by examining how the interventions can meet the users’ needs, how the user interacts with the intervention, and the practical challenges, which might be unique among users. Studies primarily used qualitative approaches such as focus groups, semistructured interviews, open-ended questions, and participatory workshops [43,82]. Qualitative analysis was applied to field notes and reflective logs to explore the users’ phenomenological insights, offering a deep understanding of their lived experiences with the intervention. For example, Shkel et al [43] integrated qualitative findings with quantitative measures, including crowdsourced support and health questionnaires, to contextualize user feedback on areas for improvement in their intervention. Valentine et al [82] used a phenomenological approach to explore the lived experiences of patients through open-ended questions and reflective logs to document user interactions and perceptions. Insight revealed themes related to engagement, emotional support, and practical challenges, providing implementable insights to improve the design of the intervention.

Third, for implementation and feasibility, 2 studies evaluated the clinical outcomes and practical considerations of DMHIs, such as usability in health care settings and implementation barriers. To achieve this, the studies adopted a mixed-method approach. For instance, van Orden et al [72] and Mayer et al [76] examined the feasibility of integrating DMHIs into health care workflows while evaluating their effectiveness. Thus, reflecting a proposed balance between evaluating clinical outcomes and addressing possible real-world challenges that might occur during or with their use.

Fourth, for longitudinal tracking, 3 longitudinal studies sought to evaluate the long-term impact of the interventions on the users. These studies combined quantitative symptom tracking with qualitative user feedback to assess the ongoing engagement and intervention durability of the interventions (eg, [75,83]). The nature of this evaluation approach provides insight into the long-term impact and engagement with DMHIs over time, thereby providing researchers with foresight on what features, functions, or interaction qualities might be redundant over time.

The studies illustrated a range of methodological approaches. For example, 4 studies used RCTs to assess the effectiveness of the interventions, while others opted for retrospective observational designs, which allow evaluation in real-world settings. Further, 2 studies used mixed-method approaches, combining qualitative and quantitative data to balance measurable outcomes with rich, contextual insights. Finally, 2 studies focused exclusively on qualitative methods to explore the user experiences.

First, for quantitative approaches, the evaluation focus often influences the approach or method used by the researchers in assessing the DMHIs. For example, quantitative studies reportedly use experimental designs and standardized measurement tools to evaluate effectiveness, feasibility, and long-term impacts of the DMHIs. RCT experiments were particularly prominent in studies evaluating effectiveness and symptom tracking, emphasizing their role in establishing relationships and intervention efficacy. For example, Kerber et al [75] used baseline, postintervention, and follow-up assessments to evaluate a self-guided transdiagnostic app, measuring its effects on mental health symptoms and quality of life over time [43,73,84]. Similarly, RCTs were used to demonstrate intervention efficacy. Nonrandomized approaches, such as retrospective observational studies, were also adopted in evaluating real-world applications of the DMHIs. Venkatesan et al [80] used this approach by using retrospective data to assess a therapy-supported app for depression and anxiety, combining standardized questionnaires with observational data to measure engagement, symptom improvement, and effectiveness. As highlighted in the studies, standardized tools were consistently used in quantitative studies to ensure reliable, feasible, and comparable outcomes by measuring symptom severity, well-being, and functional outcomes. These tools include the PHQ-8 and PHQ-9 (Patient Health Questionnaire), which were frequently used to measure depression severity, as seen in studies by Cuijpers et al [73], Mayer et al [76], and Venkatesan et al [80]. The GAD-7 (Generalized Anxiety Disorder-7 Scale) was similarly used by Venkatesan et al [80] and Klein et al [85] to quantify anxiety symptoms. Some studies adopted broader well-being metrics, such as the WHO-5 (World Health Organization Well-Being Index) and the WHODAS-12 (World Health Organization Disability Assessment Schedule), as demonstrated by Cuijpers et al [73] to provide a more comprehensive understanding of the intervention’s effects on overall functioning and symptom reduction. Klein et al [85] incorporated specialized tools such as e-PASS and the Kessler-6 scale to evaluate anxiety treatments and assess a range of *DSM-IV-TR* (*Diagnostic and Statistical Manual of Mental Disorders, Fourth Edition, Text Revision*) disorders across multiple self-help e-therapy programs. In addition to these tools, some studies focused on service-level and user-level metrics to assess their intervention at scale by analyzing real-world usage and outcomes. Harty et al [74], for instance, conducted a retrospective observational study of a supported digital CBT service offered by the national health service. Routine outcome monitoring and repeated assessments further contributed to the evaluation of DMHIs by tracking changes in symptoms, satisfaction, and engagement over time. These approaches provided insights into both the immediate efficacy and the long-term impact of interventions.

Second, for qualitative approaches*,* qualitative methodologies are frequently used in DMHI studies, especially those focused on assessing the user experience with the interventions. These methodologies provide insights into the users’ needs, perceptions, and the contextual relevance of the DMHIs. Thereby providing subjective nuances that influence the development, implementation, and usefulness of these interventions, insights that are not fully captured by quantitative methods. A total of 2 studies [43,82] of the 11 studies focused on evaluation, exclusively adopted qualitative approaches to evaluate the user experience with DMHI designed for multiple mental health challenges. Further, 3 qualitative approaches emerged across these studies. These approaches were semistructured interviews and open-ended questions to gather detailed user feedback; field notes and reflective logs to capture contextual nuances and user behaviors; and qualitative analysis frameworks such as phenomenological approaches to explore deeper insights into the impact and usability of the DMHIs. For instance, Shek et al [43] combined an RCT with qualitative methodologies to evaluate a web-based intervention, *Overcoming Thoughts,* designed for anxiety and depression. By conducting semistructured interviews with 23 participants, the study collected detailed insights into the intervention’s acceptability, usability, and perceived impact. Similarly, Valentine et al [82] conducted a qualitative evaluation of a long-term social media-based intervention for young people experiencing first-episode psychosis. This study used open-ended questions, field notes, and reflective logs to gain phenomenological insights into participants’ experiences with the intervention. The study highlighted the intervention’s ability to meet user needs, its practical challenges, and the broader contextual factors influencing its effectiveness, by focusing on the lived experiences of users.

Third, mixed-method approaches, some studies integrated both quantitative and qualitative methods to provide a more comprehensive evaluation of the DMHIs. For instance, Mayer et al [76] used a mixed-methods approach, combining quantitative assessments such as the PHQ-9 with qualitative feedback through daily evaluation sheets. This combined approach not only quantifies clinical outcomes but also captures daily user experiences and engagement levels, which are relevant for assessing the practicality and acceptability of the intervention. Van Orden et al [72] performed a naturalistic retrospective cohort study comparing the effects of a need-driven DMHI for patients with depression or anxiety disorders with traditional face-to-face treatment. A total of 3 cases were further analyzed to demonstrate the expressions of personalization in individual treatment. These expressions of personalization include interaction patterns between therapist and patients, registration possibilities, and treatment progress patterns. KLANT (short) questionnaire and a standardized self-report outcome questionnaire (OQ-45.2) were used for repeated measurement of patient progress during the treatment process (quantitative), alongside qualitative data collection to explore both the clinical outcomes and the personal experiences of participants.

### Recommended Implementation Strategies

We identified 2 recommended strategies for implementation from the studies included in the evaluation papers. First, integration into health systems and clinical workflows. Cuijpers et al [73] recommended integrating DMHIs through health authorities, local medical centers, and employers to increase accessibility and user reach. Graham et al [84] highlighted the need for future research to develop and evaluate strategies for integrating DMHIs into clinical workflows. Additionally, Kerber et al [75] suggested that a longer intervention and follow-up period may be necessary to foster and measure changes in trust among mental health providers.

Second, personalized or tailored experiences. Studies emphasized the importance of personalizing DMHIs based on users’ clinical profiles or needs [43,72,76]. For example, Shkel et al [43] and Pozuelo et al [86] recommended offering privacy options and guidance on how to use the platform effectively based on individual clinical needs. Additionally, implementing peer support features and demographic-specific features could provide a more tailored experience for users, particularly for older adults [83] and young people [84].

## Discussion

### Principal Findings

This scoping review aims to address the following research questions: (1) What design principles and evaluation approaches are used in DMHIs? (2) How are these principles, approaches, or strategies applied in the DMHI development process? The findings reveal that multiple design principles and evaluation approaches are used in DMHIs, with emphasis on user-centered design and stakeholder involvement as design principles. The evaluation approaches focus on context-dependent factors—process and outcome measures, as well as interaction qualities such as the effectiveness of interventions and user experiences and perceptions. These approaches can be related to the process-outcome measure phenomenon [87]. For instance, the studies explore the constructs to change during or in between the intervention use (ie, process) and their association with subsequent change in symptoms or users’ behavior or experiences after using the intervention (ie, outcome) [88,89]. These insights highlight the importance of integrating both methodological rigor and user engagement in the design, evaluation, and implementation of DMHIs.

A summary of the key findings includes:

1. Design principles: common practices include iterative user-centered development, expert inclusion, usability testing, specifying the design elements (event-based and minimal interface design, information visualization), and user tracking with feedback.
2. Evaluation approaches: methods vary based on intervention type, study method, and aim. A range of methods—including quantitative, qualitative, and mixed-method approaches—were used to assess DMHIs, each with distinct applications and insights. These include quantitative methods such as RCTs to emphasize intervention efficacy; qualitative approaches focusing on user experience; and mixed-method evaluations to bridge these aspects.
3. Recommendations for implementation: while the included studies did not explicitly report on implementation strategies, several recommendations were made to support the integration of DMHIs into the health care ecosystem. These include incorporating peer support features, personalizing experiences based on users’ clinical profiles and demographic-specific needs, and integrating DMHIs into health systems and clinical workflows through partnerships with health authorities, local medical centers, and employers to expand user reach and improve access.

In the following sections, we recommend 8 guidelines for DMHI projects, which detail the best practices for design, evaluation, and implementation. Each guideline [50] is supported by findings from the review and existing literature, highlighting their practical implications and the strategies that aim to enhance intervention effectiveness, user engagement, and integration into health care systems.

### Recommended Guidelines for DMHI Projects

#### Overview

DMHIs offer a promising approach to delivering accessible and effective mental health support. To optimize their effectiveness and adoption, it is important to implement evidence-based design, evaluation, and implementation strategies. We, therefore, present a structured set of guidelines to ensure that DMHIs are user-centered, evaluated, and integrated into existing health care systems.

#### Guideline 1: Prioritize End User or Patient Involvement

User involvement in DMHI design should be prioritized, whether in an informative, consultative, or fully collaborative manner [90]. Additionally, studies could integrate user feedback through iterative usability testing, ensuring interventions align with user needs and expectations. Furthermore, iterative development processes commonly adopted in the reviewed studies were often framed as user-centered approaches. Agile methodologies, prototyping, usability testing, and qualitative feedback were frequently used by the reviewed studies to refine interventions. These interactive approaches, therefore, seem to represent best practices that future research could continue to implement to ensure DMHIs align with user needs.

#### Guideline 2: Optimize Expert Involvement Without Replacing End Users

In cases of limited user access or low retention, some studies substituted end users with experts. While expert involvement provided valuable insights into user needs and vulnerabilities [45], it was often unclear whether the final interventions were based on explicit user requirements or expert suggestions. While experts bring extensive knowledge, relying on them as proxies risks introducing design bias, potentially misaligning the intervention with user needs [91]. Thus, ensuring that DMHIs are evaluated by end users helps in establishing a more truthful understanding of their effectiveness and relevance [92]. Likewise, the contribution of future research would increase when they transparently report the stakeholders involved, detailing when and how they contributed [47]. We, therefore, recommend that evaluation with end users is included in the decision-making process or during validation of the intervention. Ensuring that the tool meets the actual needs of the target population before mass production of these tools.

#### Guideline 3: Provide Clear Justification for Design Decisions or Choices

Future studies could explicitly state the rationale behind design choices. Reporting on decision-making processes will improve transparency, reproducibility, and best practice dissemination in DMHI research. Future research could also attempt to report their design activities based on the recommendations stated by Vial et al [47], such as explicitly stating, defining, and providing justification for the applied design principles, as well as explaining how stakeholders were involved in the design or development process. For instance, studies noted challenges in user recruitment and engagement, often leading to low retention rates. This aligns with the law of attrition by Eysenbach [93], which examines factors influencing digital intervention engagement. Limited user involvement during design can weaken iterative processes, leading to interventions that fail to meet user needs, thereby reducing adoption and retention rates.

Although studies reported the design principles they used, the rationale for selecting or adapting these principles was often unclear. Understanding the implications of design choices could help future designers understand whether to apply them to their situation, as modifications based on constraints (eg, limited user access) may impact outcomes. Therefore, we encourage future research to explicitly justify design decisions and explore how adapting principles affects intervention effectiveness and user engagement.

#### Guideline 4: Clearly Define and Report Applied Design Principles

We identified that studies did not explicitly report their design principles as principles or used varied terminology, even when describing similar activities or applications. This lack of consistency makes it difficult to understand and replicate the approaches used for DMHI design. While absolute standardization may not be necessary, researchers should strive for greater consistency in naming and defining design principles. Explicitly stating and defining design principles will help researchers and practitioners better understand and compare approaches across studies. For instance, while some studies [77,86] adopt a user-centered approach, others describe similar methods but use different terminology. Some studies refer to participatory design, while others mention cocreation or user involvement without clearly differentiating these concepts [78,81]. Inconsistencies such as these could make it difficult for researchers—especially those from non-HCI or clinical backgrounds—to interpret and apply design practices effectively. Future research should strive to define and justify the design principles they use, ensuring transparency and consistency. To address this, we recommend aligning terminology with established frameworks such as ISO 9241 HCD principles or providing clear definitions when introducing design terms.

#### Guideline 5: Ensure Stakeholder Involvement in the Design Process and DMHI Integration

Collaboration with diverse stakeholders, including mental health professionals, researchers, and policymakers, is important for integrating DMHIs into real-world settings [73,94]. Studies are encouraged to proactively identify potential barriers to both user adoption and implementation during the design phase to ensure more engaging, effective interventions [71]. This could be achieved by careful incorporation of iterative stakeholder involvement and engagement during all stages of the DMHI development and implementation. Stakeholder involvement not only enhances intervention design by providing feedback that can elucidate perceptions about deficits in the current system but also provides relevant insight for troubleshooting challenges earlier [95]. Additionally, they could facilitate smoother implementation by helping researchers and designers identify initial barriers to the implementation or use of DMHIs [94,95], consequently saving time and money.

Studies have shown that stakeholder perspectives and acceptance influence intervention relevance and feasibility [96-98]. Engaging stakeholders in design and development could foster familiarity and confidence in DMH technologies, reducing resistance to their adoption and integration in real-life settings. Therefore, future design projects should strive for transparency in documenting the extent of stakeholder involvement and clarifying their roles.

To further strengthen the design process, cocreation and co-design could be actively integrated into DMHI development. Cocreation involves stakeholders throughout all stages of DMHI development, promoting collaborative problem-solving. Co-design focuses on engaging stakeholders in designing solutions for specific, predefined challenges [99]. Both approaches enhance user engagement and ensure that DMHIs include diverse perspectives to shape the design process and are tailored to real-world needs.

#### Guideline 6: Prioritize Personalization to Enhance Engagement and Effectiveness

DMHIs could benefit from tailored experiences based on user clinical profiles and preferences while ensuring privacy options and effective platform guidance [43,74,86]. Personalization options, such as adaptive interfaces and personalized content, have the potential to improve user engagement and long-term adherence. Future research should aim to investigate how to integrate these considerations into DMHI design, as personalization can help address key barriers such as acceptability, credibility, and technical feasibility, ultimately improving intervention uptake and effectiveness [77].

#### Guideline 7: Address Challenges in Evaluation by Selecting Appropriate Methodologies

Evaluating DMHIs requires methodological and analytical choices that influence research outcomes. Two key factors influence these evaluations: the evaluation focus (effectiveness, user experience, implementation feasibility, and longitudinal tracking) and the methodological approach (qualitative, quantitative, or mixed methods). Understanding these factors helps establish a comprehensive framework for assessing DMHI efficacy, usability, and long-term impact. Studies should align evaluation approaches with the intervention’s goals. While RCTs remain the gold standard for evaluating clinical efficacy, minimizing selection bias, and confounding factors [46,100]. However, the rapidly evolving nature of DHIs presents challenges for traditional RCT structures, which are often lengthy and rigid [101,102]. By the time an RCT is completed, the intervention may already be outdated [39]. Additionally, as DMHIs are typically personalized for individual users, between-group comparisons in RCTs may fail to capture personalization’s impact on effectiveness [103].

To address these limitations, future research could explore alternative evaluation models that balance methodological rigor with the flexibility required for digital health research. For instance, researchers could consider alternative methods such as microrandomized trials or mixed-method approaches to capture real-world usability, feasibility, and long-term effectiveness.

#### Guideline 8: Focus on Real-World Implementation Strategies

Integrating DMHIs into existing health care ecosystems requires partnerships with health care authorities, local medical centers, and employers. Addressing potential barriers such as user trust, accessibility, and system integration could improve adoption and sustainability [54,73].

### Future Research

While the included studies adopted human-centered approaches such as user-centered design, the justifications of their design choices were not explicitly stated in their report. Therefore, the outcomes reported in these studies do not paint the full picture of their research activity. We suggest that future studies provide design justifications for a better understanding of the design and implementation process of DMHIs. Doing so might assist in translating these outcomes and justifications into effective design principles for DMHIs.

We also see a potential for future multidisciplinary collaboration, given that the review studies highlighted collaborative approaches with experts to design and evaluate the DMHIs. However, studies also reported on using experts as proxies in cases where access to the users or patients is limited. This is an opportunity for studies that successfully recruit and collaborate with users to report on how to engage and involve these groups, as well as report on the collaboration process with experts and the implications of this on DMHIs projects. Consequently, leading the way to better understanding interprofessional dynamics in multi- or interdisciplinary collaborations for DMH. We also welcome more research that focuses on establishing design principles for collaboration with users among other stakeholders, given the issues surrounding access to users and low retention with intervention usage. Additionally, we suggest that future research report on their use of existing design principles and adopt the principle of including different perspectives into the design, evaluation, and implementation of DMHIs.

Finally, while there have been notable advances in the development, adoption, and use of DMHIs, there are considerable contextual differences across health systems, levels of technology adoption, and cultural attitudes toward mental health. Overlooking these differences may introduce bias into research samples and intervention designs, as the majority of studies in this review are from Western countries and consequently reflect predominantly Western perspectives. Moreover, limited digital literacy and a lack of cultural sensitivity pose barriers to the accessibility and effectiveness of DMHIs [104]. For instance, individuals experiencing mental health challenges in contexts where stigma persists and digital literacy is low are vulnerable to digital exclusion. This exclusion could limit their ability to access, navigate, engage, and benefit from certain digital interventions, as well as obtain timely mental health information [105]. To address these challenges, future research could develop strategies that enable designers to be more attentive to cultural perceptions of mental health conditions, and the digital habits of their intended users. Integrating such sensitivity into design practices could contribute to reducing inequalities in health care access and empower individuals with mental health needs to make informed decisions and achieve improved health outcomes. Furthermore, research could also explore ways to bridge the digital divide and examine which established empirical insights and design principles can be generalized across contexts, and which remain culture- or system-specific. Addressing these dimensions, cultural sensitivity, digital equity, and generalization of design knowledge could foster the development of DMHIs that are inclusive, user-centered, adaptable, and empowering, while safeguarding privacy and fostering full participation on a global scale [106].

### Limitations

To our knowledge, this is the first scoping review to explore the design principles and the evaluation approaches for digital interventions for mental health care promotion and well-being. However, there are some limitations that must be accounted for when interpreting the findings of this scoping review. We searched a limited selection of databases, and numerous studies were screened; relevant studies might have been missed. Only peer-reviewed papers were included, published data, and excluded gray literature; therefore, some relevant literature may have been missed. Studies explicitly mentioning design principles, evaluation approaches, and especially implementation strategies were especially difficult to identify, which explains there being no implementation studies included in the review. Another limitation is that we did not appraise the included studies for quality, given that this scoping review aimed to provide an overview of a large and diverse body of literature on the design principles and evaluation approaches currently used in DMHI.

### Final Remarks

This scoping review explored the design principles, evaluation approaches, and implementation strategies of DMHIs to address our research questions. The results highlight the practice of applying HCD approaches such as user-centered design, stakeholder involvement, and the integration of diverse evaluation methods in ensuring effectiveness and user satisfaction. Although implementation strategies were not explicitly detailed in the reviewed studies, recommendations were identified to support the integration of DMHIs into health care systems and real-life settings. Therefore, we encourage future research that focuses on improving these strategies for the mental health context and exploring best practices for scaling DMHIs to maximize their accessibility, usability, and impact on mental health outcomes. Furthermore, we believe that more emphasis should be placed on inter- or multidisciplinary collaboration and adaptive innovation, as this could help realize their potential.

## Supplementary material

10.2196/77038 Multimedia Appendix 1 A detailed overview of the search string used for this scoping review.

10.2196/77038 Multimedia Appendix 2 Full -text assessment and list of excluded studies.

10.2196/77038 Multimedia Appendix 3 The data extraction form used for this scoping review.

10.2196/77038 Multimedia Appendix 4 Overview of the metadata and population characteristics of the included studies.

10.2196/77038 Multimedia Appendix 5 Overview of the digital intervention characteristics of the included studies.

10.2196/77038 Checklist 1 PRISMA-ScR checklist.

## Abbreviations

CBT: cognitive behavioral therapy
DHI: digital health intervention
DMH: digital mental health
DMHI: digital mental health intervention
*DSM-IV-TR*: *Diagnostic and Statistical Manual of Mental Disorders, Fourth Edition, Text Revision*
GAD-7: Generalized Anxiety Disorder-7 Scale
HCD: human-centered design
HCI: human-computer interaction
ISO: International Organization for Standardization
JBI: Joanna Briggs Institute
PHQ: Patient Health Questionnaire
PRISMA: Preferred Reporting Items for Systematic Reviews and Meta-Analyses
PRISMA-ScR: Preferred Reporting Items for Systematic Reviews and Meta-Analyses Extension for Scoping Reviews
RCT: randomized controlled trial
WHO-5: World Health Organization Well-Being Index
WHODAS-12: World Health Organization Disability Assessment Schedule
