# Supplementary material for: Designing and Evaluating Digital Mental Health Interventions: Scoping Review
Source: JMIR Ment Health. 2026 Apr 29;13:e77038. doi: 10.2196/77038 (PMC13128068; doi:10.2196/77038)
Supplement: Multimedia Appendix 1 [file mental-v13-e77038-s002.docx]

Search String

TITLE-ABS-KEY ( "digital mental health intervention" OR "digital mental health application*" OR "digital mental health intervention*" OR "digital mental health technolog*" OR "e-MHealth intervention*" OR "e-Mental health intervention*" OR "e-MHealth technolog*" OR " e-Mental health technolog*" OR "internet-based mental health intervention*" OR "e-MHealth application*" OR "e-mental health application" OR "internet-based mental health application*" )

AND TITLE-ABS-KEY ( "design principles" OR "principles for designing" OR "design framework" OR "development" OR "implementation" OR "strategies" OR "guidelines" OR "creation" OR "design*" OR "develop*" )

AND TITLE-ABS-KEY ( "evaluation" OR "review" OR "analysis" OR evaluat* )

AND TITLE-ABS-KEY ( "*mental" OR "psych*" OR "mood" OR "suic*" OR "well being" OR "wellbeing" OR "well-being" OR "stress" OR "distress" OR "mental disorder*" OR "mental illness*" OR "phobia" )

AND (

LIMIT-TO ( DOCTYPE , "ar" )

OR LIMIT-TO ( DOCTYPE , "cp" )

)

AND (

LIMIT-TO ( LANGUAGE , "English" )

)
