# Supplementary material for: Designing and Evaluating Digital Mental Health Interventions: Scoping Review
Source: JMIR Ment Health. 2026 Apr 29;13:e77038. doi: 10.2196/77038 (PMC13128068; doi:10.2196/77038)
Supplement: Multimedia Appendix 3 [file mental-v13-e77038-s003.docx]

| **List of excluded studies (N=18)** |
| --- |
| Reason: Unclear description of the design and evaluation process of a DMHI including clear description of design principles and evaluation approaches used (N=10)  [^1–10^] |
| Reason: Focused or designed for conditions (users) other than mental conditions as a primary condition (N=5)  [^11–15^] |
| Reason: No full paper or unable to access to the full paper including study protocol (N=3)  [^16–18^] |

**References**

1. Forman-Hoffman VL, Pirner MC, Flom M, et al. Engagement, Satisfaction, and Mental Health Outcomes Across Different Residential Subgroup Users of a Digital Mental Health Relational Agent: Exploratory Single-Arm Study. *JMIR Form Res*. 2023;7(1). doi:10.2196/46473

2. Jonathan GK, Abitante G, McBride A, et al. LiveWell, a smartphone-based self-management intervention for bipolar disorder: Intervention participation and usability analysis. *J Affect Disord*. 2024;350:926-936. doi:10.1016/j.jad.2024.01.099

3. Becker J, Kreis A, Schorch T, et al. Adherence and effectiveness of an emotion-based psychodynamic online self-help during and after inpatient and day-care psychotherapy: Results of a naturalistic study. *Front Psychiatry*. 2023;14. doi:10.3389/fpsyt.2023.1027118

4. Zech JM, Johnson M, Pullmann MD, et al. An Integrative Engagement Model of Digital Psychotherapy: Exploratory Focus Group Findings. *JMIR Form Res*. 2023;7. doi:10.2196/41428

5. Beltzer ML, Daniel KE, Daros AR, Teachman BA. Changes in Learning From Social Feedback After Web-Based Interpretation Bias Modification: Secondary Analysis of a Digital Mental Health Intervention Among Individuals With High Social Anxiety Symptoms. *JMIR Form Res*. 2023;7. doi:10.2196/44888

6. Graham AK, Kwasny MJ, Lattie EG, et al. Targeting subjective engagement in experimental therapeutics for digital mental health interventions. *Internet Interv*. 2021;25. doi:10.1016/j.invent.2021.100403

7. Rickhi B, Kania-Richmond A, Moritz S, et al. Evaluation of a spirituality informed e-mental health tool as an intervention for major depressive disorder in adolescents and young adults - a randomized controlled pilot trial. *BMC Complement Altern Med*. 2015;15(1). doi:10.1186/s12906-015-0968-x

8. Graham AK, Lattie EG, Powell BJ, et al. Implementation strategies for digital mental health interventions in health care settings. *American Psychologist*. 2020;75(8):1080-1092. doi:10.1037/amp0000686

9. Raphiphatthana B, Sweet M, Dingwall K, MacDonald A, Nagel T. The Weathering Well App, A New Low Intensity Digital Mental Health Intervention for Australian Farmers: Formative Research. Published online March 5, 2021. doi:10.21203/rs.3.rs-269174/v1

10. Lukka L, Salonen A, Vesterinen M, Karhulahti VM, Palva S, Palva JM. The qualities of patients interested in using a game-based digital mental health intervention for depression: a sequential mixed methods study. *BMC Digital Health*. 2023;1(1). doi:10.1186/s44247-023-00037-w

11. Choi SK, Bruehlman-Senecal E, Green A, Lavra J, Bauermeister J. Patterns of engagement in digital mental health intervention for LGBTQ+ youth: a latent profile analysis. *Front Digit Health*. 2023;5. doi:10.3389/fdgth.2023.1254929

12. Gleeson J, Lin A, Koval P, et al. Moderated Online Social Therapy for Carers of Early Psychosis Clients in Real-World Settings: Cluster Randomized Controlled Trial. *JMIR Ment Health*. 2023;10(1). doi:10.2196/47722

13. Bäuerle A, Jahre L, Teufel M, et al. Evaluation of the E-Mental Health Mindfulness-Based and Skills-Based “CoPE It” Intervention to Reduce Psychological Distress in Times of COVID-19: Results of a Bicentre Longitudinal Study. *Front Psychiatry*. 2021;12. doi:10.3389/fpsyt.2021.768132

14. Gabrielli S, Rizzi S, Bassi G, et al. Engagement and effectiveness of a healthy-coping intervention via chatbot for university students during the COVID-19 pandemic: Mixed methods proof-of-concept study. *JMIR Mhealth Uhealth*. 2021;9(5). doi:10.2196/27965

15. Morris RR, Kouddous K, Kshirsagar R, Schueller SM. Towards an artificially empathic conversational agent for mental health applications: System design and user perceptions. *J Med Internet Res*. 2018;20(6). doi:10.2196/10148

16. Andrews B, Klein B, Corboy D, McLaren S, Watson S. Video Chat Therapist Assistance in an Adaptive Digital Intervention for Anxiety and Depression: Reflections From Participants and Therapists. *Prof Psychol Res Pr*. 2023;54(6):418-429. doi:10.1037/PRO0000527

17. Dirmaier J, Liebherz S, Sänger S, Härter M, Tlach L. Psychenet.de: Development and process evaluation of an e-mental health portal. *Inform Health Soc Care*. 2016;41(3):267-285. doi:10.3109/17538157.2015.1008486,

18. Thevathasan L, Fairley L, Phillips C, et al. Coproducing multilingual conversational scripts for a mental wellbeing chatbot - where healthcare domain experts become chatbot designers. *European Psychiatry*. 2022;65(S1):S293-S293. doi:10.1192/J.EURPSY.2022.748
