# Supplementary material for: Designing and Evaluating Digital Mental Health Interventions: Scoping Review
Source: JMIR Ment Health. 2026 Apr 29;13:e77038. doi: 10.2196/77038 (PMC13128068; doi:10.2196/77038)
Supplement: Multimedia Appendix 4 [file mental-v13-e77038-s004.docx]

| **Concept** | **Definition** |
| --- | --- |
| **Study Characteristics** |  |
| Author | The first author of the study. |
| Year of Publication | The year in which the study was published. |
| Country of publication | The country where the study was published. |
| Type of publication | The medium in which the study was published (e.g. conference proceedings, journal, thesis). |
| Study design | The research method that the study used to collect the data. |
| Study aim | What the study aimed to find out |
| Study setting | Whether clinical or non-clinical study |
| Study type | How the data is collected or analysed (whether qualitative, quantitative, experimental etc.) |
| **Population characteristics** |  |
| Number of participants | Number of people who participated in the study. |
| Age (mean) | The (average) age of participants. |
| **Intervention characteristics** |  |
| Intervention name | Name of the DMHI. |
| Intervention platform | Where you can access the DMHI |
| Intervention purpose | What it is that the DMHI aims to achieve (therapy, education, counselling, self-management, screening, diagnosing) |
| Intervention type | The platform in which the intervention was implemented (i.e. mobile application, web-based, or multimodal platform). |
| Targeted disorder | What mental health challenge would the DMHI support |
| Design Features | The features included in the DMHI |
| **Design principles** |  |
| Reported design principles | What is the design principle applied in the study |
| Application of the design principles | How was the principle applied |
| **Evaluation approaches** |  |
| Evaluation methods and tools | What is the evaluation approach used in the study; these include the tools used for the evaluation process |
| Outcome measured | What is the outcome that the study measured?  experiences, effectiveness, usability, satisfaction… |
| Data collection techniques | How was the data collected measured |
| **Findings** |  |
| Results of the study | The main findings for relevant outcomes |
| Recommendations for future work | The recommendations stated in the study for future development, adoption, or use of their work |
| Recommended implementation strategies | The implementation strategies recommended for the digital interventions for mental healthcare. |
