# Supplementary material for: Designing and Evaluating Digital Mental Health Interventions: Scoping Review
Source: JMIR Ment Health. 2026 Apr 29;13:e77038. doi: 10.2196/77038 (PMC13128068; doi:10.2196/77038)
Supplement: Multimedia Appendix 5 [file mental-v13-e77038-s005.docx]

| Author (year)^ID^ | Country | Publication type | Study design  (as reported) | Sample size | Age  (as reported) | Study Setting |
| --- | --- | --- | --- | --- | --- | --- |
| Xiang et al, (2023)^1^ | USA | Journal | Qualitative | >10 | >60 | Non-clinical |
| Shkel et al, (2023)^2^ | USA | Journal | Qualitative | 23 | 34 mean | Clinical |
| van Orden et al (2022)^3^ | Netherlands | Journal | Exploratory naturalistic retrospective cohort study | >4222  DMHI users = (169)  Traditional treatment = (4053)  Personalised care cases = (3) | 35.7 mean  (DMHI users)  40.9  (traditional users) | Clinical |
| Cuijpers et al, (2022)^4^ | Lebanon | Journal | Single-blind, two-arm pragmatic RCT | 680 | 27 mean | Clinical |
| Harty et al, (2023)^5^ | Ireland | Journal | Quantitative | 2255 | 18- 65+ | Clinical |
| Kerber et al, (2023)^6^ | Germany | Journal | Retrospective Observational study | 1045  Intervention group = (523)  Control group = (522) | 18-77 | Clinical |
| Mayer et al, (2022)^7^ | Germany | Journal | RCT | 110 participants (41 patients and 69 healthy controls) | 30.47 mean | Clinical |
| Burchert et al, (2019)^8^ | Germany  Sweden  Egypt | Journal | Mixed method | 128 | 18> | Non-clinical |
| Stegemann et al, (2013)^9^ | Germany | Conference proc. | Cross sectional observational study | NR | NR | Non-clinical |
| Geraghty et al, (2016)^10^ | UK | Journal | Qualitative | 20 | 51 mean | Non-clinical |
| Venkatesan et al, (2020)^11^ | USA | Journal | Qualitative | 323 | 36.42 | Clinical |
| Ferguson et al, (2021)^12^ | USA | Conference proc. | Qualitative | >14  7782 | NR | Non-clinical |
| Valentine et al, (2020)^13^ | Australia | Journal | Quantitative Retrospective Observational | 12 | 19-28 | Clinical |
| Gould et al, (2021)^14^ | USA | Journal | Mixed method | 50 | 40-81 | Clinical |
| Graham et al, (2020)^15^ | USA | Journal | Qualitative | 146 | 42.3 mean | Clinical |
| Klein et al, (2011)^16^ | Australia | Journal | Quantitative | 225 | >18 | Clinical |
| Pozuelo et al, (2023)^17^ | South Africa Uganda | Journal | RCT | 160 | 15-19 | Clinical |

1. Xiang X, Kayser J, Ash S, et al. Web-Based Cognitive Behavioral Therapy for Depression Among Homebound Older Adults: Development and Usability Study. *JMIR Aging*. 2023;6(1). doi:10.2196/47691

2. Shkel J, Green G, Le S, et al. Understanding Users’ Experiences of a Novel Web-Based Cognitive Behavioral Therapy Platform for Depression and Anxiety: Qualitative Interviews From Pilot Trial Participants. *JMIR Form Res*. 2023;7. doi:10.2196/46062

3. van Orden ML, Kraaijeveld JC, Spijker AT, et al. Evaluating the first results of a need-driven digital mental health intervention for depression and anxiety; an exploratory study. *Clinical eHealth*. 2022;5:44-51. doi:10.1016/j.ceh.2022.06.002

4. Cuijpers P, Heim E, Ramia JA, et al. Guided digital health intervention for depression in Lebanon: Randomised trial. *Evid Based Ment Health*. 2022;25(e1):E34-E40. doi:10.1136/ebmental-2021-300416

5. Harty S, Enrique A, Akkol-Solakoglu S, et al. Implementing digital mental health interventions at scale: one-year evaluation of a national digital CBT service in Ireland. *Int J Ment Health Syst*. 2023;17(1). doi:10.1186/s13033-023-00592-9

6. Kerber A, Beintner I, Burchert S, Knaevelsrud C. Effects of a Self-Guided Transdiagnostic Smartphone App on Patient Empowerment and Mental Health: Randomized Controlled Trial. *JMIR Ment Health*. 2023;10(1). doi:10.2196/45068

7. Mayer G, Hummel S, Oetjen N, et al. User experience and acceptance of patients and healthy adults testing a personalized self-management app for depression: A non-randomized mixed-methods feasibility study. *Digit Health*. 2022;8. doi:10.1177/20552076221091353

8. Burchert S, Alkneme MS, Bird M, et al. User-centered app adaptation of a low-intensity e-mental health intervention for Syrian refugees. *Front Psychiatry*. 2019;10(JAN). doi:10.3389/fpsyt.2018.00663

9. Stegemann SK, Lehr D, Berking M, Funk B, Ebenfeld L. *Development of a Mobile Application for People with Panic Disorder as Augmentation for an Internet-Based Intervention*.; 2014. http://www.fearfighter.com/

10. Geraghty AWA, Muñoz RF, Yardley L, Mc Sharry J, Little P, Moore M. Developing an unguided internet-delivered intervention for emotional distress in primary care patients: Applying common factor and person-based approaches. *JMIR Ment Health*. 2016;3(4). doi:10.2196/mental.5845

11. Venkatesan A, Rahimi L, Kaur M, Mosunic C. Digital cognitive behavior therapy intervention for depression and anxiety: Retrospective study. *JMIR Ment Health*. 2020;7(8). doi:10.2196/21304

12. Ferguson C, Lewis R, Wilks C, Picard R. The Guardians: Designing a Game for Long-term Engagement with Mental Health Therapy. *IEEE Conference on Computatonal Intelligence and Games, CIG*. 2021;2021-August. doi:10.1109/COG52621.2021.9619026

13. Valentine L, McEnery C, O’Sullivan S, Gleeson J, Bendall S, Alvarez-Jimenez M. Young people’s experience of a long-term social media-based intervention for first-episode psychosis: Qualitative analysis. *J Med Internet Res*. 2020;22(6). doi:10.2196/17570

14. Gould CE, Carlson C, Alfaro AJ, Chick CF, Bruce ML, Forman-Hoffman VL. Changes in Quality of Life and Loneliness Among Middle-Aged and Older Adults Participating in Therapist-Guided Digital Mental Health Intervention. *Front Public Health*. 2021;9. doi:10.3389/fpubh.2021.746904

15. Graham AK, Greene CJ, Kwasny MJ, et al. Coached mobile app platform for the treatment of depression and anxiety among primary care patients: A randomized clinical trial. *JAMA Psychiatry*. 2020;77(9):906-914. doi:10.1001/jamapsychiatry.2020.1011

16. Klein B, Meyer D, Austin DW, Kyrios M. Anxiety online-A virtual clinic: Preliminary outcomes following completion of five fully automated treatment programs for anxiety disorders and symptoms. *J Med Internet Res*. 2011;13(4). doi:10.2196/jmir.1918

17. Pozuelo JR, Moffett BD, Davis M, et al. User-Centered Design of a Gamified Mental Health App for Adolescents in Sub-Saharan Africa: Multicycle Usability Testing Study. *JMIR Form Res*. 2023;7:e51423. doi:10.2196/51423
