# Supplementary material for: Designing and Evaluating Digital Mental Health Interventions: Scoping Review
Source: JMIR Ment Health. 2026 Apr 29;13:e77038. doi: 10.2196/77038 (PMC13128068; doi:10.2196/77038)
Supplement: Checklist 1 [file mental-v13-e77038-s006.docx]

| Author (year)ID | Name | Purpose | Type | Targeted Disorder | Design features |
| --- | --- | --- | --- | --- | --- |
| Xiang et al, (2023)^1^ | Empower@Home | Prevent and reduce depressive symptoms | Web-based | Depression | -9 web-based lessons, each featuring didactic content, in-session exercises, motivational quotes, and an engaging animated story driven by human characters.  -Mood self-check by filling out the Patient Health Questionnaire-9  (PHQ-9) to track their symptoms.  -Provider dashboard that allows providers to review patients’ progress, enabling them to readily access easily digestible data for quality improvement and evaluation  purposes. |
| Shkel J et al, (2023)^2^ | Overcoming Thoughts | Providing Cognitive Behavioural Therapy | Web-based | Anxiety & Depression | 2 exercises involving cognitive restructuring and behavioural experimentation practices. |
| van Orden et al (2022)^3^ | NiceDay | Provide need driven treatment | Mobile & Web-based | Anxiety & Depression | -Chat function  -Videoconferencing  -Diary and feeling journal  -Feedback sessions  -Thought recording  -Tasks and events planning  -Treatment related monitoring for therapists |
| Cuijpers et al, (2022)^4^ | Step by Step | Treat and provide psychoeducation and training in behavioural activation. | Mobile & Web-based | Depression | -Stress management  -Gratitude exercise  -Positive self- talk  -Social support and relapse prevention.  -Illustrative narrative  -Emails or phone-based reminders or notifications |
| Harty et al, (2023)^5^ | SilverCloud | Develop and increase understanding  of the relationship between one’s thoughts, feelings, and  behaviours, alongside information about coping strategies and therapeutic techniques | Web-based | Mental Well-being | -Weekly reminder emails  -item-9 of the Patient  Health Questionnaire-9),  Generalised Anxiety Disorder-2  -Interactive media and tools, including quizzes, videos, activities, and personal stories |
| Kerber et al, (2023)^6^ | Mind Doc | Help users  by  self-monitoring, automated feedback and  psychological courses and exercises. | Mobile-based | Mental Well-being | -Regular self-monitoring, automated feedback, and psychological courses and exercises  -Biweekly feedback and encouragement  -Push notifications  -Information on how to access mental health care.  -Free and premium (paid) features |
| Mayer et al, (2022)^7^ | SELFPASS: Self-administered Psycho Therapy SystemS | Improve self-management of patients  with depression on the basis of an individualized daily mood score by delivering a daily monitoring of depressive symptoms and daily interventions to support the patients | Mobile & Web-based | Depression | -Cognitive behavioural therapy approaches: journal keeping, behavioural activation, thought stop and cognitive restructuring  -Mood-related questions  -Reminders for completing daily self-assessment |
| Burchert et al, (2019)^8^ | Step by Step | Developed by the WHO to address depression, based on evidence-based psychological interventions to reduce psychological distress and improve functioning in communities affected by adversity, using a scalable and adaptable approaches | Mobile-based | Depression | -Messaging  -Storytelling through illustrated educative narratives and interactive exercises presented by a fictional main character and a fictional health professional  -Trained supervior/e-helper  -Websites, apps, audio, video or books |
| Stegemann et al, (2013)^9^ | GET.ON PAPP | To explore the potential of a combined mobile application and internet-based intervention, which integrates, provides, and organises treatment and information for people with panic disorder with and without agoraphobia. | Mobile & Web-based | Panic disorder with and without agoraphobia. | -A diary that covers the self-monitoring aspect and  -an exposure-guide that supports people in performing exposure-exercises.  -colours for each panic-related question.  -Photo upload or taking function  -text input  -daily summaries of frequency and intensity of panic attacks and exposure exercise performance  -fixed schedule diary entry" |
| Geraghty et al, (2016)^10^ | Healthy Paths through Stress | To support primary care  patients in reducing emotional distress with unguided internet -delivered intervention. | Internet-based | Emotional Distress | -Common factor model: explanation of the user’s symptoms, therapeutic rationale, therapeutic techniques for ameliorating symptoms, use of techniques and skills in daily life, improvement to the user’s skilfulness and understanding  -Text and audio content  -Hi-resolution nature images  -3 paths to a range of therapeutic techniques  -12 content modules, 4 coordination modules  -Logic models |
| Venkatesan et al, (2020)^11^ | Vida Health | Digital health program for mental health and cardiometabolic conditions  Connects adults living with mild to moderate depression or anxiety with a licensed therapist | Mobile-based | Depression, Anxiety | For users:  -Tailored digital content paired with remote therapy and health coaching with licensed therapists.  -Audio, video, and text-format lessons, activities and practices based on CBT.  -Multiple choice lesson options, checklists, and free text  -Review concepts through reading, listening to audio practices, or watching videos.  For Therapists:  -30 mins (phone or video) Weekly consultations: mood, set weekly goals, preparations for lessons and activities  -Review the completed lessons weekly and review strategies for applying the concepts and skills that had been covered.  -Generate personalised treatment plan and Wellness -Recovery Action plan. |
| Ferguson et al, (2021)^12^ | The Guardians: Unite the Realms | Help plays improve their mental health by performing real-world challenges. | Mobile-based | Mental well-being | -Gaming features such as rewards to be used immediately: pets, virtual currency, cosmetic items, tools  - Pet collection strategy game  -Adaptive real-world and mission activities with end goal of freeing pets from evil  -Reminder notifications  -Reflection on mood after performing actions  -Customisation of pets |
| Valentine et al, (2020)^13^ | Horyzons | Designed to reinforce each other, creating a flow  for the young person with first episode psychosis between the social and therapy elements” | Web-based | Psychosis | -Closed, secure, and private digital platform  -Structured web-based pathways for engaging in therapy sessions, supported by clinical moderators for accountability.  -Web-based chat for direct communication with clinical moderators privately.  -Social network (newsfeed) for public interaction, allowing users to post, comment, share experiences, and support each other.  -Clinical moderators who provide support and oversight through web-based chats and phone calls.  -Assigned clinical moderator: Each participant has a dedicated moderator to monitor engagement and progress throughout the intervention.  -Peer interaction through therapeutic social network where users can connect with others, share experiences, and receive support.  -Peer interaction through group discussions facilitated by peer moderators, providing a space for focused conversation and community-building.  -Reinforced integration of the platform’s digital and human functions. These features are designed to complement each other, creating a flow between therapy and social interaction for the users. |
| Gould et al, (2021)^14^ | Meru Health program | To manage and decrease symptoms in middle age and older adults struggling with loneliness, depression, and anxiety by improving their quality of life | Mobile-based | Loneliness, Depression, Anxiety | -Social support through the therapist-moderated discussion among the group participants.  -Informational videos and guided practices  -Therapist and anonymous peer support  -A dashboard for therapist to oversee the MHP progress of a  patient. The therapist interacts with the patients by sending  weekly  -Informational emails, asynchronous secure messaging  within the app, and conducting phone/video calls, therapist-moderated discussions |
| Graham et al, (2020)^15^ | IntelliCare | Supporting  access to clinically focused IntelliCare apps for users with anxiety and depression. | Mobile-based | Anxiety, Depression | -A suite 5 clinically focused of mobile apps.  -Library of psychoeducational  material,  -Weekly symptom assessment  -Coach support:  Short message service text messaging  -Online dashboard providing information on participant’s app use and weekly symptom assessment score |
| Klein et al, (2011)^16^ | AnxietyOnline | To increase access to  mental health services by reducing the common obstacles and to provide consumers with choice in regard to treatment, as is the case for real-world settings | Web-based | Generalized anxiety disorder  Panic disorder with or without agoraphobia  Obsessive–compulsive disorder  Posttraumatic stress disorder  Social anxiety disorder | - Open access psychoeducational website: Provides information and resources for users.  - e-PASS: An online psychological assessment and referral system.  - e-Therapy programs: Five interactive, automated 12-module self-help or therapist-assisted treatment programs with multimedia materials (audio, video, animations) and online activities (self-monitoring, quizzes, journal writing).  - Downloadable resources: Includes PDFs with worksheets, audio transcripts, and monitoring forms.  - e-Therapist/CBT training: Online programs for e-therapists and a portal for healthcare practitioners. |
| Pozuelo et al, (2023)^17^ | Kuamsha app | To deliver behavioural activation therapy for users in depression in an engaging way. | Mobile-based | Depression | -Narrative game format: Uses storytelling and immersive narratives to engage users.  -Real-life exercises: Incorporates homework activities to practice learned skills.  -Mood tracking: Helps adolescents recognize the link between mood and behaviour.  -Push notifications: Reminds users to report progress on homework tasks.  -Game design elements: Includes character personalization, a point system, and engaging mini-games.  -Password protection: Ensures secure access to the app.  -Offline mode: Allows use of the app without an internet connection.  -Onboarding process: Guides users through the app’s features.  -Emergency button: Refers adolescents at risk of suicide to appropriate support. |

**References**

1. Xiang X, Kayser J, Ash S, et al. Web-Based Cognitive Behavioral Therapy for Depression Among Homebound Older Adults: Development and Usability Study. *JMIR Aging*. 2023;6(1). doi:10.2196/47691

2. Shkel J, Green G, Le S, et al. Understanding Users’ Experiences of a Novel Web-Based Cognitive Behavioral Therapy Platform for Depression and Anxiety: Qualitative Interviews From Pilot Trial Participants. *JMIR Form Res*. 2023;7. doi:10.2196/46062

3. van Orden ML, Kraaijeveld JC, Spijker AT, et al. Evaluating the first results of a need-driven digital mental health intervention for depression and anxiety; an exploratory study. *Clinical eHealth*. 2022;5:44-51. doi:10.1016/j.ceh.2022.06.002

4. Cuijpers P, Heim E, Ramia JA, et al. Guided digital health intervention for depression in Lebanon: Randomised trial. *Evid Based Ment Health*. 2022;25(e1):E34-E40. doi:10.1136/ebmental-2021-300416

5. Harty S, Enrique A, Akkol-Solakoglu S, et al. Implementing digital mental health interventions at scale: one-year evaluation of a national digital CBT service in Ireland. *Int J Ment Health Syst*. 2023;17(1). doi:10.1186/s13033-023-00592-9

6. Kerber A, Beintner I, Burchert S, Knaevelsrud C. Effects of a Self-Guided Transdiagnostic Smartphone App on Patient Empowerment and Mental Health: Randomized Controlled Trial. *JMIR Ment Health*. 2023;10(1). doi:10.2196/45068

7. Mayer G, Hummel S, Oetjen N, et al. User experience and acceptance of patients and healthy adults testing a personalized self-management app for depression: A non-randomized mixed-methods feasibility study. *Digit Health*. 2022;8. doi:10.1177/20552076221091353

8. Burchert S, Alkneme MS, Bird M, et al. User-centered app adaptation of a low-intensity e-mental health intervention for Syrian refugees. *Front Psychiatry*. 2019;10(JAN). doi:10.3389/fpsyt.2018.00663

9. Stegemann SK, Lehr D, Berking M, Funk B, Ebenfeld L. *Development of a Mobile Application for People with Panic Disorder as Augmentation for an Internet-Based Intervention*.; 2014. http://www.fearfighter.com/

10. Geraghty AWA, Muñoz RF, Yardley L, Mc Sharry J, Little P, Moore M. Developing an unguided internet-delivered intervention for emotional distress in primary care patients: Applying common factor and person-based approaches. *JMIR Ment Health*. 2016;3(4). doi:10.2196/mental.5845

11. Venkatesan A, Rahimi L, Kaur M, Mosunic C. Digital cognitive behavior therapy intervention for depression and anxiety: Retrospective study. *JMIR Ment Health*. 2020;7(8). doi:10.2196/21304

12. Ferguson C, Lewis R, Wilks C, Picard R. The Guardians: Designing a Game for Long-term Engagement with Mental Health Therapy. *IEEE Conference on Computatonal Intelligence and Games, CIG*. 2021;2021-August. doi:10.1109/COG52621.2021.9619026

13. Valentine L, McEnery C, O’Sullivan S, Gleeson J, Bendall S, Alvarez-Jimenez M. Young people’s experience of a long-term social media-based intervention for first-episode psychosis: Qualitative analysis. *J Med Internet Res*. 2020;22(6). doi:10.2196/17570

14. Gould CE, Carlson C, Alfaro AJ, Chick CF, Bruce ML, Forman-Hoffman VL. Changes in Quality of Life and Loneliness Among Middle-Aged and Older Adults Participating in Therapist-Guided Digital Mental Health Intervention. *Front Public Health*. 2021;9. doi:10.3389/fpubh.2021.746904

15. Graham AK, Greene CJ, Kwasny MJ, et al. Coached mobile app platform for the treatment of depression and anxiety among primary care patients: A randomized clinical trial. *JAMA Psychiatry*. 2020;77(9):906-914. doi:10.1001/jamapsychiatry.2020.1011

16. Klein B, Meyer D, Austin DW, Kyrios M. Anxiety online-A virtual clinic: Preliminary outcomes following completion of five fully automated treatment programs for anxiety disorders and symptoms. *J Med Internet Res*. 2011;13(4). doi:10.2196/jmir.1918

17. Pozuelo JR, Moffett BD, Davis M, et al. User-Centered Design of a Gamified Mental Health App for Adolescents in Sub-Saharan Africa: Multicycle Usability Testing Study. *JMIR Form Res*. 2023;7:e51423. doi:10.2196/51423
